# Temporal changes in post-bariatric nutritional deficiency anemia: a propensity score-matched analysis of 2015–2017 versus 2022–2023 cohorts

**DOI:** 10.3389/fnut.2026.1820503

**Published:** 2026-07-02

**Authors:** I-Wen Chen, Chia-Li Kao, Yi-Chen Lai, Ying-Jen Chang, Kuo-Chuan Hung

**Affiliations:** 1 Department of Anesthesiology, Chi Mei Hospital, Liouying, Tainan, Taiwan; 2 Department of Anesthesiology, E-Da Hospital, I-Shou University, Kaohsiung, Taiwan; 3 Department of Anesthesiology, Chi Mei Medical Center, Tainan, Taiwan

**Keywords:** bariatric surgery, gastric bypass, iron deficiency anemia, nutritional anemia, sleeve gastrectomy

## Abstract

**Background:**

Metabolic and bariatric surgery (MBS) is associated with postoperative nutritional deficiencies, yet whether temporal shifts in surgical practice and healthcare delivery have influenced the risk of nutritional anemia remains unclear. We compared the 2-year incidence of postoperative nutritional anemia between patients who underwent MBS between 2015 and 2017 and 2022–2023.

**Methods:**

Using the TriNetX Global Collaborative Network, we conducted a propensity score–matched cohort study of adult patients undergoing laparoscopic sleeve gastrectomy or Roux-en-Y gastric bypass. The primary outcome was the 2-year cumulative incidence of nutritional anemia, assessed from 90 to 730 days postoperatively. The secondary outcomes included iron deficiency anemia (IDA), vitamin B12 deficiency anemia, other anemia, all-cause hospitalization, and emergency department visits.

**Results:**

After 1:1 propensity score matching, 13,010 patients were included in each cohort. Compared with the early cohort (2015–2017), the late cohort (2022–2023) had a significantly higher risk of nutritional anemia (6.61% vs. 5.30%; hazard ratio [HR] 1.34, 95% confidence interval [CI] 1.22–1.49; *p* < 0.001), including iron deficiency anemia (HR 1.36, *p* < 0.001) and vitamin B12 deficiency anemia (HR 1.32, *p* = 0.025). No significant difference was observed for other anemia (HR 1.05, *p* = 0.386). The risk of all-cause hospitalization was higher in the late cohort (HR 1.57, *p* < 0.001), whereas emergency department visits did not differ significantly. Sensitivity and subgroup analyses yielded consistent findings for the primary outcome and iron deficiency anemia, although the association with vitamin B12 deficiency anemia was not uniformly significant across all models.

**Conclusion:**

In this observational study, the 2022–2023 cohort had a higher risk of coded or clinically detected postoperative nutritional anemia than earlier cohorts. Given the modest absolute risk difference and a diagnosis-code–based outcome, this finding should be interpreted as a higher burden of recognized anemia diagnoses rather than definitive evidence of increased biological anemia incidence. These results support adherence to existing postoperative nutritional monitoring recommendations rather than a more intensive surveillance schedule.

## 1 Introduction

Metabolic and bariatric surgery (MBS) is an established therapeutic strategy for severe obesity and is associated with significant improvements in metabolic control, cardiovascular risk factors, and renal outcomes (1–4). However, the anatomical and physiological alterations induced by these procedures predispose patients to nutritional deficiencies, among which anemia remains one of the most prevalent and clinically significant complications (5–8). Iron deficiency anemia and vitamin B12 deficiency anemia are particularly common following Roux-en-Y gastric bypass and sleeve gastrectomy, driven by reduced absorptive surface area, diminished gastric acid secretion, and altered dietary intake (9–11). Current clinical practice guidelines (12, 13) emphasize structured perioperative nutritional assessment, routine postoperative biochemical monitoring, and lifelong micronutrient supplementation after bariatric procedures, yet anemia and related micronutrient deficiencies remain common during the first postoperative year after MBS. Large-scale U. S. data have demonstrated increasing rates of postoperative nutritional deficiencies over time (14), and long-term prospective observations have confirmed that micronutrient deficiencies may accumulate despite supplementation (15). However, prior studies (14, 15) primarily described overall temporal trends across extended periods and did not directly compare distinct contemporary surgical eras under differing healthcare contexts.

Despite the advances in bariatric care guidelines, postoperative nutritional surveillance and long-term adherence to supplements remain challenging. Over the past decade, surgical volume has expanded, sleeve gastrectomy has become dominant, patient comorbidity profiles have evolved, and perioperative follow-up patterns have been influenced by broader healthcare system changes, including disruptions during the COVID-19 pandemic (16, 17). Whether these shifts have translated into improved nutritional safety or altered the risk of postoperative nutritional anemia in real-world practice remains unclear.

To address this gap, we conducted a propensity score–matched cohort study using the TriNetX Global Collaborative Network to compare the 2-year incidence of postoperative nutritional anemia between patients undergoing MBS in 2015–2017 and those treated in 2022–2023. By evaluating two discrete surgical eras separated by substantial healthcare system changes, we sought to determine whether contemporary bariatric care is associated with a differential risk of postoperative nutritional anemia.

## 2 Methods

### 2.1 Study design and cohort identification

We conducted a retrospective propensity score–matched cohort study using the TriNetX Global Collaborative Network, a federated health research platform that aggregates de-identified electronic health record data from healthcare organizations across the United States. The TriNetX database has been widely utilized in large-scale observational and cohort studies in multiple clinical and epidemiological research settings (18–21). The study protocol was approved by the Institutional Review Board of Chi Mei Medical Center, which waived the requirement for informed consent in accordance with the regulations for observational research (IRB No. 11403-E01).

Adult patients (≥18 years old) who underwent primary laparoscopic sleeve gastrectomy (CPT 43775) or laparoscopic Roux-en-Y gastric bypass (CPT 43644) were eligible for inclusion. The date of the first qualifying procedure was defined as the index date. Two temporal cohorts were constructed: an early cohort (surgery between January 1, 2015, and December 31, 2017) and a late cohort (surgery between January 1, 2022, and December 31, 2023). The 2015–2017 window was chosen to provide an early pre-pandemic comparison period while minimizing pandemic-related under-follow-up. These two periods were selected to capture potential changes in clinical practice, nutritional monitoring protocols, and surgical volume before and after the intervention period, which included the COVID-19 pandemic and evolving bariatric care guidelines, while allowing sufficient follow-up duration for both cohorts.

Patients were excluded if they were diagnosed with nutritional anemia (ICD-10-CM D50–D53) or other anemia (ICD-10-CM D64) recorded before surgery or within 90 days of the index date. This washout window was applied to minimize the misclassification of pre-existing or perioperative anemia as incident postoperative events. Patients with advanced chronic kidney disease (CKD stages 4–5 or end-stage renal disease; ICD-10-CM N18.4, N18.5, N18.6) documented before the index date were also excluded to reduce confounding from severe renal dysfunction, which may independently predispose patients to anemia. To ensure adequate follow-up, the included patients were required to have at least one healthcare encounter recorded between 3 months and 2 years after the index date.

### 2.2 Propensity score matching

To reduce measured confounding between the two temporal cohorts, 1:1 propensity score matching was performed using a greedy nearest-neighbor algorithm without replacement. Propensity scores were estimated via logistic regression with the cohort period (early vs. late) as the dependent variable. Covariates included demographic characteristics, comorbidities, procedure type, baseline medication use, and baseline laboratory parameters; the full list of variables is provided in Supplementary Table S1. Procedure type and baseline laboratory values were included directly in the propensity score model. These variables were selected *a priori* based on clinical relevance and their potential association with postoperative nutritional anemia risk and temporal differences in patient characteristics. A caliper width of 0.1 standard deviations of the logit of the propensity score was applied. Covariate balance was assessed using standardized mean differences (SMDs), with values below 0.1 considered indicative of adequate balance.

### 2.3 Outcome assessment

The primary outcome was the 2-year cumulative incidence of nutritional anemia, assessed within a landmark window beginning 90 days after surgery and extending to 2 years after surgery. Nutritional anemia was defined as a composite outcome using ICD-10-CM codes D50–D53, including iron deficiency anemia (D50), vitamin B12 deficiency anemia (D51), folate deficiency anemia (D52), and other nutritional anemias (D53) (Supplementary Table 1). Secondary outcomes included the 2-year risks of selected nutritional anemia subtypes, specifically iron deficiency anemia (D50) and vitamin B12 deficiency anemia (D51), along with other anemia (ICD-10-CM D64), all-cause hospitalization, and emergency department visits. We additionally evaluated outcomes within a 1- to 2-year postoperative window because nutritional anemia after MBS often develops gradually and may be more reflective of long-term nutritional status and supplement adherence rather than early postoperative recovery. Patients were censored at outcome occurrence, death, or end of follow-up.

### 2.4 Sensitivity and subgroup analyses

Three sensitivity analyses evaluated the robustness of the primary findings: Model I restricted the analysis to patients who underwent surgery at academic medical centers; Model II was limited to patients with documented iron or vitamin B12 laboratory measurements during the 3-month to 2-year postoperative window; and Model III restricted the cohort to female patients. Because postoperative laboratory testing is itself a post-baseline process, Model II was interpreted as a supportive subgroup analysis rather than a definitive adjustment for surveillance bias. Subgroup analyses were stratified by the type of surgery (sleeve gastrectomy vs. gastric bypass), with interaction terms included to assess effect modification.

### 2.5 Additional analysis

To address concerns related to the ICD-10-CM coding transition and early-cohort window selection, we performed alternative early-era window analyses. In these analyses, the late cohort remained defined as patients undergoing MBS in 2022–2023, whereas the early cohort was redefined using two alternative windows: 2016–2017 and 2017–2019. Both alternative early-era windows were selected to reduce the potential influence of the initial ICD-10-CM transition. The 2016–2017 window avoided the 2015 transition while remaining close to the original early era, whereas the 2017–2019 window provided a later pre-pandemic surgical cohort but may have had follow-up partly affected by the COVID-19 period. We therefore retained both windows to evaluate whether the findings were sensitive to early-cohort definition, coding-transition effects, and pandemic-era follow-up patterns.

To further evaluate differential detection of anemia, we examined follow-up hemoglobin testing frequency in the alternative early-era window analyses. Hemoglobin testing was selected as the most direct laboratory indicator of opportunity for anemia detection, because anemia diagnosis requires assessment of hemoglobin status. In contrast, follow-up encounters may reflect postoperative complications or healthcare utilization rather than surveillance alone, and iron or vitamin B12 testing reflects micronutrient evaluation but does not necessarily indicate anemia assessment. Therefore, we summarized, by era, the proportion of patients with at least one hemoglobin measurement and the number of hemoglobin measurements among tested patients during the 90-day to 2-year postoperative window. We did not use crude testing counts across the full matched cohort as the primary metric because patients without recorded tests may represent loss to follow-up, limited healthcare contact, or absence of clinical indication, rather than lower surveillance intensity alone.

### 2.6 Statistical analysis

Continuous variables are presented as means with standard deviations, and categorical variables as frequencies with percentages. Because this study was based on electronic health record data, missing laboratory values were not imputed, and analyses were conducted using available data within the propensity score matching framework. Because this study used a federated electronic health record database, individual-level loss to follow-up could not be directly measured. Follow-up time was defined based on available healthcare encounters, and patients were censored at the time of outcome occurrence, death, or the end of the observation period. Associations between the cohort period and time-to-event outcomes were estimated using Cox proportional hazards regression models and reported as hazard ratios (HRs) with 95% confidence intervals (CIs). Statistical significance was defined as a two-sided *p*-value less than 0.05. Because these analyses were exploratory and hypothesis-generating, we did not apply formal multiple-comparison adjustment, and the related findings should be interpreted cautiously. All analyses were performed using the integrated analytical tools of the TriNetX platform.

## 3 Results

### 3.1 Patient selection and baseline characteristics

**Figure 1:**
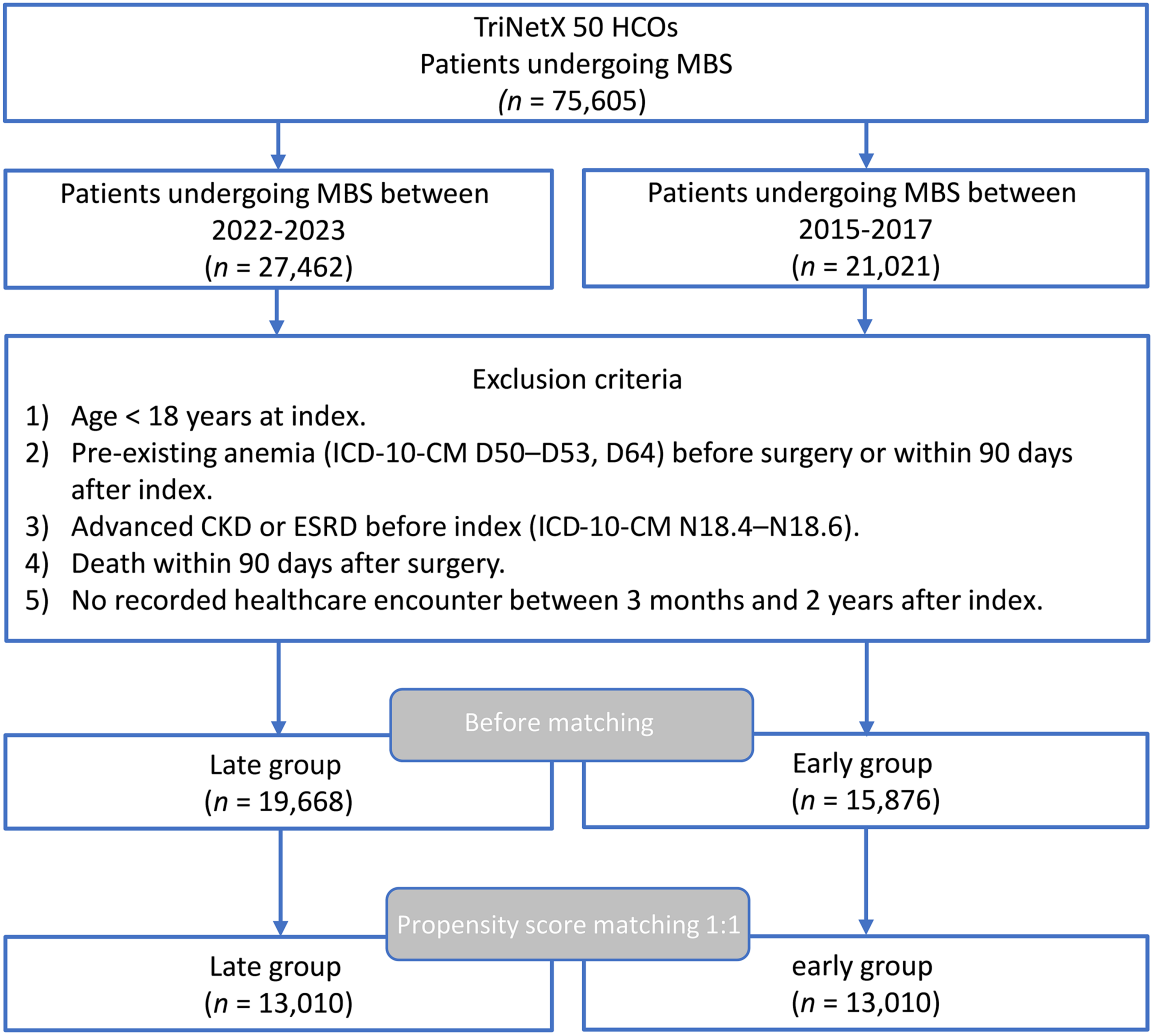
Flow diagram of cohort selection from the TriNetX Research Network. MBS, metabolic and bariatric surgery; CKD, chronic kidney disease; ESRD, end-stage renal disease; PSM, propensity score matching.

**Figure 2:**
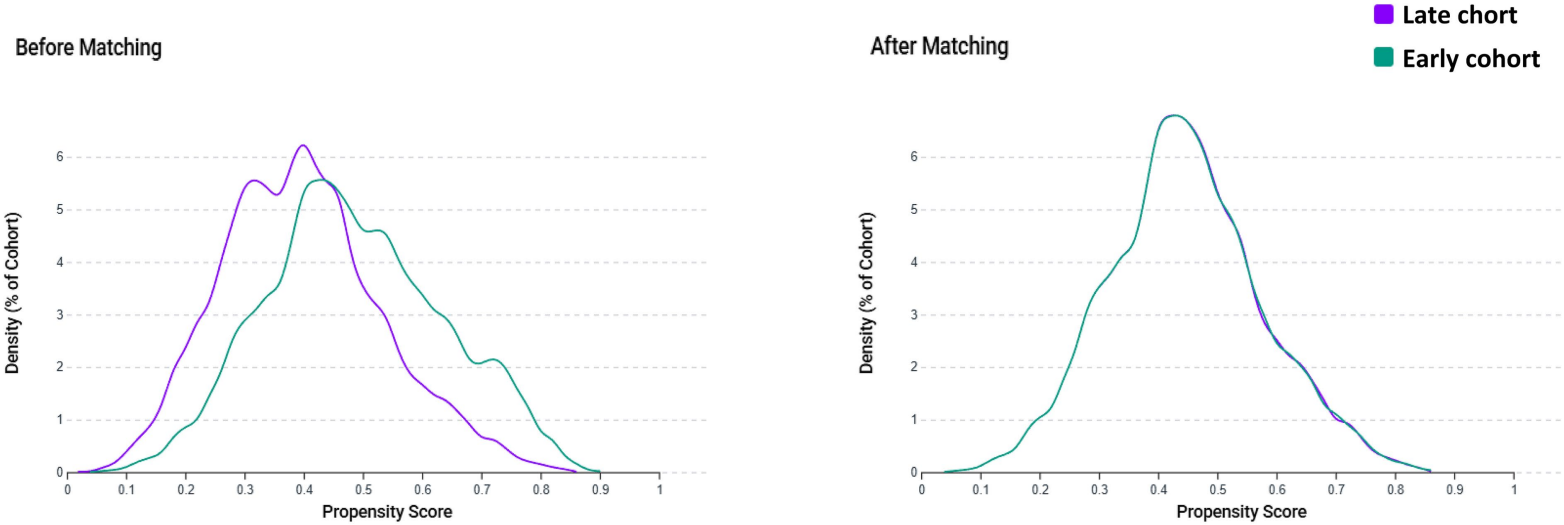
Distribution of propensity scores before and after 1:1 propensity score matching. Density plots of estimated propensity scores showing the distribution of estimated propensity scores for the late cohort (2022–2023) and early cohort (2015–2017) before and after matching. Before matching, the two cohorts demonstrated partial separation in propensity score distributions. After 1:1 nearest-neighbor matching with a caliper of 0.1 standard deviations of the logit of the propensity score, substantial overlap was achieved, indicating improved covariate balance between groups.

From the TriNetX Research Network, 19,668 patients in the late cohort (2022–2023) and 15,876 patients in the early cohort (2015–2017) met the initial eligibility criteria (Figure 1). After applying the exclusion criteria and 1:1 propensity score matching, 13,010 patients remained in each group. Following matching, all baseline covariates achieved adequate balance, with standardized mean differences below 0.1 across all variables (Table 1). The distribution of propensity scores demonstrated substantial overlap between the two cohorts after matching, whereas partial separation was observed before matching (Figure 2). The matched cohorts were similar with respect to age (43.8 ± 11.7 vs. 43.8 ± 11.8 years), proportion of female patients (80.1% vs. 79.9%), prevalence of super obesity (BMI ≥ 50 kg/m^2^; 30.2% vs. 30.5%), and the distribution of comorbidities including hypertension, diabetes mellitus, obstructive sleep apnea, and chronic kidney disease. Baseline laboratory parameters, preoperative medication use, and surgical procedure type were also well balanced between the groups. Data availability for baseline laboratory variables varied across patients. The availability of key laboratory and anthropometric variables before and after propensity score matching is summarized in Supplementary Table 2.

**Table 1:** Baseline characteristics of patients undergoing metabolic and bariatric surgery.

Variables	Before matching			After matching		
	Late group (*n* = 19,668)	Early group (*n* = 15,876)	SMD	Late group (*n* = 13,010)	Early group (*n* = 13,010)	SMD
Age, years	42.7 ± 11.6	44.5 ± 11.9	0.153	43.8 ± 11.7	43.8 ± 11.8	0.002
BMI ≥ 50 kg/m^2^	6,148 (31.3)	4,560 (28.7)	0.055	3,931 (30.2)	3,971 (30.5)	0.007
Female	16,034 (81.5)	12,506 (78.8)	0.069	10,422 (80.1)	10,400 (79.9)	0.004
White	12,737 (64.8)	11,626 (73.2)	0.184	9,175 (70.5)	9,177 (70.5)	0.000
Black or African American	4,208 (21.4)	2,897 (18.2)	0.079	2,550 (19.6)	2,577 (19.8)	0.005
Other Race	1,111 (5.6)	380 (2.4)	0.166	400 (3.1)	362 (2.8)	0.017
Asian	110 (0.6)	69 (0.4)	0.018	57 (0.4)	65 (0.5)	0.009
Comorbidities						
Factors influencing health status and contact with health services	17,173 (87.3)	12,927 (81.4)	0.163	11,008 (84.6)	10,990 (84.5)	0.004
Essential (primary) hypertension	10,887 (55.4)	9,584 (60.4)	0.102	7,609 (58.5)	7,618 (58.6)	0.001
Obstructive sleep apnea (adult) (pediatric)	10,852 (55.2)	8,598 (54.2)	0.020	7,113 (54.7)	7,118 (54.7)	0.001
Diabetes mellitus	5,654 (28.7)	5,227 (32.9)	0.091	4,038 (31.0)	4,011 (30.8)	0.004
Diseases of liver	5,576 (28.4)	2,890 (18.2)	0.242	2,693 (20.7)	2,738 (21.0)	0.009
Neoplasms	3,130 (15.9)	2,575 (16.2)	0.008	2,103 (16.2)	2,127 (16.3)	0.005
Nicotine dependence	2,103 (10.7)	1,165 (7.3)	0.117	1,079 (8.3)	1,062 (8.2)	0.005
Major depressive disorder, recurrent	2,251 (11.4)	1,054 (6.6)	0.168	1,004 (7.7)	1,019 (7.8)	0.004
Ischemic heart diseases	1,141 (5.8)	1,060 (6.7)	0.036	830 (6.4)	824 (6.3)	0.002
Other chronic obstructive pulmonary disease	552 (2.8)	554 (3.5)	0.039	413 (3.2)	397 (3.1)	0.007
Chronic kidney disease (CKD)	545 (2.8)	392 (2.5)	0.019	347 (2.7)	338 (2.6)	0.004
Cerebrovascular diseases	293 (1.5)	274 (1.7)	0.019	201 (1.5)	212 (1.6)	0.007
Alcohol related disorders	368 (1.9)	228 (1.4)	0.034	201 (1.5)	203 (1.6)	0.001
Laboratory data						
eGFR ≥60 mL/min/1.73 m^2^	17,582 (89.4)	13,077 (82.4)	0.203	11,419 (87.8)	11,462 (88.1)	0.010
Albumin ≥3.5 g/dL	17,236 (87.6)	11,747 (74.0)	0.352	10,723 (82.4)	10,756 (82.7)	0.007
Hemoglobin≥12	17,226 (87.6)	12,082 (76.1)	0.301	10,839 (83.3)	10,804 (83.0)	0.007
Hemoglobin A1c ≥ 7%	2,209 (11.2)	1844 (11.6)	0.012	1,528 (11.7)	1,539 (11.8)	0.003
Vitamin supplementation						
Vitamin d	8,405 (42.7)	6,313 (39.8)	0.060	5,291 (40.7)	5,332 (41.0)	0.006
Vitamin b	3,439 (17.5)	2,732 (17.2)	0.007	2,156 (16.6)	2,186 (16.8)	0.006
Iron	1,311 (6.7)	801 (5.0)	0.069	734 (5.6)	748 (5.7)	0.005
Type of surgery						
Sleeve gastrectomy	12,864 (64.6)	10,323 (65.0)	0.008	8,456 (64.9)	8,370 (64.1)	0.014
Gastric bypass	6,970 (35.4)	5,593 (35.0)	0.004	4,606 (35.1)	4,680 (35.9)	0.012

Data are presented as mean ± standard deviation or *n* (%). BMI, body mass index; eGFR, estimated glomerular filtration rate; SMD, standardized mean difference. An SMD < 0.1 indicates adequate balance between cohorts.

### 3.2 Association between time period and 2-year anemia outcomes

After propensity score matching, follow-up duration was comparable between the two cohorts. The median follow-up was 730 days in both the late and early cohorts. The interquartile range of follow-up was 24 days in the late cohort and 0 days in the early cohort. The mean follow-up duration was 648.0 ± 167.9 days in the late cohort and 693.4 ± 121.9 days in the early cohort. These findings indicate that most patients in both cohorts were followed for nearly the full 2-year observation period, although slightly greater variability in follow-up duration was observed in the late cohort.

Within the 90-day to 2-year postoperative window, the late cohort was associated with a higher risk of nutritional anemia than the early cohort (6.61% vs. 5.30%; absolute risk difference, 1.31%; HR 1.34, 95% CI 1.22–1.49, *p* < 0.001) (Table 2). This difference was primarily driven by a higher risk of iron deficiency anemia (HR 1.36, *p* < 0.001), with a smaller but statistically significant difference observed for vitamin B12 deficiency anemia (HR 1.32, *p* = 0.025). No significant difference was observed for other anemia (HR 1.05, *p* = 0.386). The late cohort was also associated with a higher risk of all-cause hospitalization (HR 1.57, *p* < 0.001), whereas no significant intergroup difference was observed for emergency department visits. The unadjusted associations between surgical era and 2-year postoperative outcomes before propensity score matching are presented in Supplementary Table 3.

**Table 2:** Association between surgical era and 2-year postoperative outcomes after propensity score matching (90-day to 2-year window).

Outcomes	Late group^†^ Events (%)	Early group^†^ Events (%)	HR (95% CI)	*p* value	Absolute risk difference
Nutritional anemia	860 (6.61%)	689 (5.30%)	1.34 (1.22–1.49)	<0.001	1.31%
IDA	706 (5.43%)	560 (4.30%)	1.36 (1.22–1.52)	<0.001	1.12%
B12 deficiency anemia	145 (1.12%)	116 (0.89%)	1.32 (1.04–1.69)	0.025	0.22%
Other anemia	578 (4.44%)	595 (4.57%)	1.05 (0.94–1.18)	0.386	−0.13%
Hospitalization	2,088 (16.05%)	1,484 (11.41%)	1.57 (1.47–1.68)	<0.001	4.64%
ED visit	2,281 (17.53%)	2,385 (18.33%)	1.02 (0.96–1.08)	0.567	−0.80%

^†^*n* = 13,010 per group; HR, hazard ratio; CI, confidence interval; IDA, iron deficiency anemia; ED, emergency department.

When the analysis was restricted to the 1- to 2-year postoperative window, the associations were of greater magnitude (Table 3). The late cohort demonstrated a higher risk of nutritional anemia (HR 1.64, *p* < 0.001), iron deficiency anemia (HR 1.64, *p* < 0.001), and vitamin B12 deficiency anemia (HR 1.73, *p* = 0.001) than the early cohort.

**Table 3:** Association between surgical era and postoperative outcomes within the 1- to 2-year postoperative window after propensity score matching.

Outcomes	Late group^†^ Events^†^ (%)	Early group^†^ Events (%)	HR (95% CI)	*p* value	Absolute risk difference
Nutritional anemia	609 (4.68%)	419 (3.22%)	1.64 (1.45–1.85)	<0.001	1.46%
IDA	515 (3.96%)	353 (2.71%)	1.64 (1.43–1.88)	<0.001	1.25%
B12 deficiency anemia	92 (0.71%)	59 (0.45%)	1.73 (1.25–2.40)	0.001	0.25%
Other anemia	402 (3.09%)	394 (3.03%)	1.15 (1.00–1.32)	0.053	0.06%
Hospitalization	1,451 (11.15%)	973 (7.48%)	1.73 (1.59–1.88)	<0.001	3.67%
ED visit	1,497 (11.51%)	1,523 (11.71%)	1.10 (1.03–1.19)	0.007	−0.20%

^†^*n* = 13,010 per group; HR, hazard ratio; CI, confidence interval; IDA, iron deficiency anemia; ED, emergency department.

### 3.3 Sensitivity analyses

The primary findings remained consistent across all three sensitivity models (Table 4). In Model I (restricted to academic medical centers), Model II (restricted to patients with documented iron or B12 laboratory measurements), and Model III (restricted to female patients), the late cohort was consistently associated with a higher risk of nutritional anemia (HRs ranging from 1.33 to 1.37, all *p* < 0.001) and iron deficiency anemia (HRs ranging from 1.37 to 1.41, all *p* < 0.001). The association with vitamin B12 deficiency anemia was statistically significant in Model II (HR 1.36, *p* = 0.020) but did not reach significance in Models I and III. The association with hospitalization remained significant across all models. Model II supported the persistence of the association among patients who underwent postoperative iron or vitamin B12 testing; however, because testing occurred after surgery, this model was not considered sufficient to exclude surveillance bias.

**Table 4:** Sensitivity analyses of the association between surgical era and 2-year postoperative outcomes.

Outcomes	Model I		Model II		Model III	
	HR (95% CI)	*p* value	HR (95% CI)	*p* value	HR (95% CI)	*p* value
Nutritional anemia	1.33 (1.18–1.50)	<0.001	1.37 (1.23–1.52)	<0.001	1.34 (1.21–1.49)	<0.001
IDA	1.37 (1.21–1.55)	<0.001	1.41 (1.25–1.58)	<0.001	1.39 (1.23–1.56)	<0.001
B12 deficiency anemia	0.75 (0.49–1.16)	0.198	1.36 (1.05–1.77)	0.020	1.28 (0.98–1.68)	0.074
Other anemia	1.12 (0.99–1.28)	0.08	1.09 (0.96–1.23)	0.177	0.99 (0.88–1.13)	0.922
Hospitalization	1.65 (1.53–1.78)	<0.001	1.57 (1.46–1.70)	<0.001	1.60 (1.48–1.72)	<0.001
ED visit	0.93 (0.86–0.99)	0.033	1.07 (1.01–1.15)	0.035	1.03 (0.97–1.10)	0.311

Model I: restricted to patients treated at academic medical centers (*n* = 10,053 per group). Model II: restricted to patients with documented iron or vitamin B12 laboratory measurements during the 3-month to 2-year postoperative window (*n* = 8,714 per group). Model III: restricted to female patients (*n* = 10,357 per group). HR, hazard ratio; CI, confidence interval; IDA, iron deficiency anemia; ED, emergency department.

### 3.4 Subgroup analyses by type of surgery

In subgroup analyses stratified by surgical procedure (Table 5), the late cohort was associated with a higher risk of nutritional anemia in both the sleeve gastrectomy (HR 1.20, *p* = 0.009) and gastric bypass (HR 1.46, *p* < 0.001) subgroups, with no significant interaction (*p* = 0.056). Iron deficiency anemia showed a similar pattern, with a significant interaction, indicating a more pronounced temporal increase in the bypass subgroup (P for interaction = 0.045). Vitamin B12 deficiency anemia reached significance only in the sleeve gastrectomy subgroup (HR 1.38, *p* = 0.049). The association with hospitalization was substantially stronger in the sleeve gastrectomy subgroup (HR, 1.87) than in the bypass subgroup (HR, 1.17; P for interaction < 0.001). No significant differences were observed for emergency department visits in either subgroup.

**Table 5:** Subgroup analyses of the association between surgical era and 2-year postoperative outcomes stratified by type of surgery.

Outcomes	Sleeve gastrectomy		Bypass surgery		P for interaction
	HR (95% CI)	*p* value	HR (95% CI)	*p* value	
Nutritional anemia	1.20 (1.05–1.38)	0.009	1.46 (1.26–1.68)	<0.001	0.056
IDA	1.20 (1.03–1.40)	0.020	1.51 (1.29–1.77)	<0.001	0.045
B12 deficiency anemia	1.38 (1.00–1.90)	0.049	1.13 (0.77–1.65)	0.533	0.436
Other anemia	0.96 (0.83–1.12)	0.640	1.20 (1.01–1.42)	0.037	0.061
Hospitalization	1.87 (1.72–2.04)	<0.001	1.17 (1.05–1.31)	0.004	<0.001
ED visit	1.02 (0.94–1.10)	0.706	1.04 (0.95–1.13)	0.427	0.745

HR, hazard ratio; CI, confidence interval; IDA, iron deficiency anemia; ED, emergency department.

### 3.5 Alternative early-era window and hemoglobin-testing analyses

In the alternative early-era window analyses, the associations were attenuated compared with the primary analysis but remained significant for coded nutritional anemia and IDA (Table 6). When the early cohort was redefined as 2016–2017, the late cohort remained associated with higher risks of nutritional anemia (HR 1.18, *p* = 0.010) and IDA (HR 1.17, *p* = 0.035). Similar findings were observed using the 2017–2019 early window for nutritional anemia (HR 1.14, *p* = 0.008) and IDA (HR 1.19, *p* = 0.001). Vitamin B12 deficiency anemia and other anemia were not consistently significant. Follow-up hemoglobin testing frequency was broadly comparable between eras (Table 7), with similar proportions of tested patients and no significant difference in mean hemoglobin measurements among tested patients in either alternative-window analysis. These findings suggest that hemoglobin testing intensity was similar between eras under the alternative early-cohort definitions.

**Table 6:** Alternative early-era window analyses of the association between surgical era and 2-year postoperative outcomes.

Outcomes	Alternative early window: 2016–2017		Alternative early window: 2017–2019	
	HR (95% CI)	*p* value	HR (95% CI)	*p* value
Nutritional anemia	1.18 (1.04–1.34)	0.010	1.14 (1.03–1.25)	0.008
IDA	1.17 (1.01–1.35)	0.035	1.19 (1.07–1.32)	0.001
B12 deficiency anemia	1.30 (0.99–1.70)	0.063	1.05 (0.84–1.30)	0.682
Other anemia	0.95 (0.82–1.10)	0.493	0.96 (0.86–1.08)	0.493
Hospitalization	1.59 (1.47–1.73)	<0.001	1.38 (1.29–1.46)	<0.001
ED visit	1.12 (1.05–1.20)	0.001	1.06 (1.01–1.12)	0.016

The late cohort was defined as 2022–2023 in both analyses. In the first alternative-window analysis, the early cohort was redefined as 2016–2017 (*n* = 8,279 per group). In the second alternative-window analysis, the early cohort was redefined as 2017–2019 (*n* = 14,502 per group), thereby avoiding the initial ICD-10-CM transition period. HR, hazard ratio; CI, confidence interval; IDA, iron deficiency anemia; ED, emergency department.

**Table 7:** Follow-up hemoglobin measurement frequency according to alternative early-cohort definitions.

Analysis	Cohort	Patients with Hb measurement	Patients with Hb measurement (%)	Number of Hb measurements (mean ± SD)	*p* value
Alternative early cohort: 2016–2017	Late cohort (*N* = 8,279)	4.045	48.90%	3.13 ± 4.93	0.767
	Early cohort (*N* = 8,279)	3.946	47.70%	3.10 ± 3.97	
Alternative early cohort: 2017–2019	Late cohort (*N* = 14,502)	7.877	54.30%	3.17 ± 4.54	0.065
	Early cohort (*N* = 14,502)	7.881	54.30%	3.05 ± 3.39	

## 4 Discussion

In this propensity score–matched cohort study of over 26,000 patients who underwent metabolic and bariatric surgery, the late cohort (2022–2023) was associated with a significantly higher 2-year risk of nutritional anemia than the early cohort (2015–2017). This temporal difference was predominantly driven by iron deficiency anemia, with a smaller contribution from vitamin B12 deficiency anemia. The associations were more pronounced when the analysis was restricted to the 1- to 2-year postoperative window and remained consistent across multiple sensitivity analyses. Subgroup analyses revealed that a temporal increase in nutritional anemia was present in both sleeve gastrectomy and gastric bypass subgroups, with a more prominent association observed in the latter for iron deficiency anemia. From a clinical perspective, the absolute risk increase (1.31%) was relatively small, indicating that while the temporal trend is statistically significant at the population level, the individual-level risk increase may be modest.

Nutritional anemia is among the most prevalent and clinically consequential complications following MBS, with reported incidence rates ranging from 7 to 40%, depending on the procedure type and follow-up duration (11, 22, 23). However, prior investigations (11, 22, 23) have predominantly focused on patient-level risk factors, such as procedure type, preoperative micronutrient status, and supplementation adherence, while the potential influence of the broader surgical era on postoperative anemia risk has received little attention. Comparing outcomes across distinct time periods is clinically relevant because system-level factors, including rapid expansion of surgical volume, evolving clinical practice guidelines, changes in follow-up infrastructure, and pandemic-related disruptions in care continuity, may collectively modify the risk of nutritional complications in ways that individual-level analyses cannot capture. Our study addresses this gap by demonstrating that after rigorous propensity score matching, the 2022–2023 cohort was associated with a higher risk of nutritional anemia than the 2015–2017 cohort, an association predominantly driven by iron deficiency anemia. This finding is biologically plausible because gastric bypass can impair iron absorption by reducing gastric acid exposure and bypassing the duodenum and proximal jejunum, whereas postoperative dietary restriction and variable supplementation adherence may further contribute to iron and vitamin B12 deficiency after MBS. Consistent with prior evidence from Aarts et al. (24), anemia and related micronutrient deficiencies may emerge within the first postoperative year, underscoring the need for continued nutritional surveillance.

The findings for vitamin B12 deficiency anemia should be interpreted with caution. Although vitamin B12 deficiency anemia was statistically significant in the main analysis and Model II, the association was not consistently observed across all models. This inconsistency suggests that the vitamin B12 finding may be less robust than the findings for iron deficiency anemia, which were consistently observed across models and analyses. The variability in the vitamin B12 results may reflect differences in model specification, smaller event numbers, residual confounding, or variability in postoperative vitamin B12 supplementation and monitoring practices. Therefore, the vitamin B12 results should be interpreted as exploratory rather than definitive.

In contrast to nutritional anemia, no significant temporal differences were observed for other types of anemia between the two cohorts. This category encompasses a heterogeneous group of conditions, including chronic anemia, sideroblastic anemia, and unspecified anemia, which may be less directly related to the nutritional consequences of MBS. The absence of a significant association for this outcome may partially support the specificity of the findings, suggesting that the temporal increase appeared to be more prominent for nutritional anemia than for anemia diagnoses in general. However, because ICD-10-CM code D64 represents a heterogeneous group of anemia conditions, it may not serve as an ideal negative control outcome. Therefore, the absence of a significant association with other anemia should be interpreted cautiously and should not be considered definitive evidence against temporal changes in coding practices or detection patterns. Rather, this finding offers only limited support for the specificity of the observed association and should be interpreted in the context of the broader study limitations.

The late cohort was associated with a significantly higher risk of all-cause hospitalization, an association that was consistent across all sensitivity and subgroup analyses. Although the late cohort demonstrated a higher risk of hospitalization, this finding should be interpreted cautiously. Hospitalization is influenced by multiple system-level and healthcare utilization factors, and the observed difference may reflect broader changes in healthcare utilization patterns, admission thresholds, comorbidity burden, follow-up practices, or healthcare access rather than a direct causal effect of nutritional anemia. The COVID-19 pandemic and its aftermath may also have influenced hospitalization patterns through delayed presentations and altered care-seeking behavior (25, 26). In contrast, no meaningful temporal difference was observed for emergency department visits, suggesting that the increased hospitalization risk is unlikely to be driven simply by a general increase in acute healthcare utilization in the later period. Whether postoperative nutritional anemia directly contributes to the higher hospitalization rate or whether both outcomes share common upstream determinants cannot be established from the present analysis and merits further investigation.

Subgroup analyses revealed that a temporal increase in nutritional anemia was present in both surgical procedure types, although the magnitude of the association was numerically greater in the gastric bypass subgroup. A significant interaction was observed for iron deficiency anemia (P for interaction = 0.045), indicating that the temporal increase in risk was more pronounced in the gastric bypass subgroup than in the sleeve gastrectomy subgroup. Because gastric bypass involves bypassing the duodenum and proximal jejunum, the primary sites of iron absorption, patients who undergo this procedure are inherently more reliant on adequate supplementation and nutritional surveillance to maintain iron homeostasis (27–29). Consequently, any temporal changes in follow-up intensity, supplementation practices, or patient adherence between the two eras would be expected to disproportionately affect patients undergoing gastric bypass, thereby amplifying the temporal difference in iron deficiency anemia risk relative to patients undergoing sleeve gastrectomy. Conversely, the association between the late cohort and hospitalization was substantially stronger in the sleeve gastrectomy subgroup, with a significant interaction. This discrepancy suggests that the factors underlying the temporal increase in hospitalization may differ by procedure type and are not merely a downstream consequence of nutritional anemia. The absence of a significant interaction for nutritional anemia overall indicates that the temporal trend is a broadly shared phenomenon across both procedures, rather than being confined to a single surgical approach.

Another important consideration is whether the observed temporal difference reflects a true increase in anemia or greater detection in the later era. To address this issue, we examined hemoglobin testing frequency because hemoglobin measurement is the most direct laboratory indicator of anemia detection. In the alternative early-era window analyses, the proportions of patients with at least one hemoglobin measurement and the mean number of hemoglobin measurements among tested patients were similar between eras, suggesting that the observed signal was not clearly driven by differential repeated hemoglobin testing. We did not use total follow-up encounters as a surveillance-adjustment variable because healthcare visits may reflect postoperative complications or hospitalization rather than neutral opportunities for anemia screening. Similarly, iron and vitamin B12 measurements indicate micronutrient surveillance but do not necessarily establish anemia. Although a hemoglobin-defined anemia outcome would have been ideal, this could not be reliably implemented in TriNetX because anemia requires sex-specific hemoglobin thresholds and the platform does not allow a single outcome definition using different cutoffs by sex. Therefore, the findings are best interpreted as a small increase in coded or clinically detected nutritional anemia rather than definitive evidence of increased laboratory-confirmed anemia incidence.

The alternative early-era window analyses were performed to evaluate whether the findings were sensitive to early-cohort definition and potential ICD-10-CM transition effects. In these analyses, the effect estimates were smaller than those in the primary analysis, suggesting that early-cohort selection or coding-transition effects may have contributed to the magnitude of the original association. However, the associations with coded nutritional anemia and iron deficiency anemia persisted even when the early cohort was redefined as 2016–2017 or 2017–2019. These findings indicate that coding-transition effects may partly, but not fully, explain the observed temporal difference.

Because the exposure in this study was defined by surgical era rather than a specific intervention, the observed differences between the two cohorts may reflect broader changes in clinical practice and healthcare delivery rather than a direct effect of the later time period itself. Over the past decade, multiple system-level factors may have changed, including diagnostic coding practices, nutritional screening intensity, postoperative follow-up patterns, dietary intake, supplement prescribing practices, adherence to micronutrient supplementation, and healthcare access. Long-term evidence after gastric bypass further indicates that nutritional deficiencies may persist or evolve over time and are influenced by adherence to standardized nutritional care, supporting the clinical importance of sustained postoperative monitoring (30). These factors may influence both the true risk and the likelihood of detecting nutritional anemia and could not be fully captured within the TriNetX platform or comprehensively adjusted for in the matching process. Therefore, residual confounding remains possible, and our findings should be interpreted as observed differences between surgical eras rather than causal effects attributable to the later period.

This absolute difference corresponds to approximately one additional coded nutritional anemia diagnosis per 76 patients, suggesting a modest bedside effect rather than a large individual-level risk increase. Therefore, these findings do not by themselves justify a new or more intensive postoperative surveillance schedule. Instead, the clinical implication is to ensure consistent adherence to existing postoperative monitoring of hemoglobin, iron status, and vitamin B12, particularly in patients at higher nutritional risk.

Other limitations of this study should be considered when interpreting these findings. First, outcomes were defined using ICD-10-CM diagnostic codes rather than serial micronutrient measurements. Accordingly, this study captured clinically recognized anemia diagnoses rather than biochemical micronutrient changes, and subclinical deficiencies may have been missed. As a result, the incidence of postoperative nutritional deficiency may have been underestimated in both cohorts, and temporal differences in coding or clinical recognition may have influenced the observed associations. Although our sensitivity analysis, restricted to patients with documented laboratory measurements, yielded consistent results, residual misclassification cannot be excluded. Second, the retrospective observational design precludes causal inference; unmeasured confounders, including changes in clinical guidelines, supplementation protocols, follow-up intensity, patient socioeconomic status, and dietary habits, may have contributed to the observed associations. Third, missing baseline laboratory data are an inherent limitation of electronic health record–based studies. Because laboratory testing is not performed uniformly for all patients, missing data may reflect differences in clinical practice patterns, patient characteristics, or healthcare utilization. As a result, residual confounding related to unmeasured laboratory values may persist despite propensity score matching, and the findings should be interpreted with this limitation in mind. Fourth, certain clinically relevant variables, such as specific micronutrient serum levels, medication adherence, and body weight trajectories, were incompletely captured within the platform and could not be incorporated into the matching algorithm. Fifth, the larger effect sizes observed in the 1- to 2-year postoperative window should be interpreted with caution, as patients who remained under surveillance during this later period may represent a non-random subset with differential healthcare utilization patterns between the two cohorts. Selective retention in follow-up may therefore have contributed to the greater magnitude of the observed associations in this time window. Finally, although the late cohort had a shorter maximum available follow-up due to the more recent surgical period, the median follow-up duration after matching was 730 days in both cohorts, indicating that most patients were observed for nearly the full intended follow-up window. However, some degree of differential censoring may still exist and should be considered when interpreting time-to-event comparisons.

## 5 Conclusion

In this propensity score–matched analysis of patients undergoing MBS, the 2022–2023 cohort was associated with a higher 2-year risk of nutritional anemia, particularly iron deficiency anemia, compared with the earlier cohort. Because the absolute risk difference was modest and the outcome was diagnosis-code based, this study cannot fully separate a true increase in anemia incidence from differential detection or coding. The most appropriate clinical interpretation is that the later era showed a higher burden of recognized anemia diagnoses, supporting careful adherence to existing postoperative hemoglobin, iron, and vitamin B12 monitoring recommendations rather than a new or more intensive surveillance schedule.

## Data Availability

The datasets presented in this article are not readily available because the data used in this study were obtained from the TriNetX Global Collaborative Network. The datasets are de-identified and aggregated within the TriNetX platform and are subject to data-use agreements, institutional policies, and TriNetX access restrictions. The authors do not have permission to download, transfer, or share patient-level data. Therefore, the generated datasets are not available from the corresponding author. Researchers with appropriate authorization may access TriNetX directly under an applicable institutional agreement. Requests to access the datasets should be directed to https://live.trinetx.com.
