# Supplementary material for: Temporal changes in post-bariatric nutritional deficiency anemia: a propensity score-matched analysis of 2015–2017 versus 2022–2023 cohorts
Source: Front Nutr. 2026 Jul 2;13:1820503. doi: 10.3389/fnut.2026.1820503 (PMC13372594; doi:10.3389/fnut.2026.1820503)
Supplement: Supplementary file 1 [file Table_1.DOCX]

**Supplemental Table S1.** **Codes Used for Cohort Definition, Outcomes, and Propensity Score Matching**

| Category | Variable | Codes / Definitions |
| --- | --- | --- |
| Inclusion criteria | Bariatric surgery | CPT 43775 – Laparoscopic sleeve gastrectomy |
|  |  | CPT 43644 – Laparoscopic Roux-en-Y gastric bypass |
|  | Age | ≥18 years |
| Exclusion criteria | Pre-existing nutritional anemia | ICD-10-CM D50–D53 |
|  | Pre-existing other anemia | ICD-10-CM D64 |
|  | Advanced chronic kidney disease | ICD-10-CM N18.4, N18.5, N18.6 |
|  | Inadequate follow-up | No healthcare encounter recorded between 3 months and 2 years after surgery |
| Primary outcome | Nutritional anemia | ICD-10-CM D50–D53, including: |
|  |  | D50 – Iron deficiency anemia |
|  |  | D51 – Vitamin B12 deficiency anemia |
|  |  | D52 – Folate deficiency anemia |
|  |  | D53 – Other nutritional anemias |
| Secondary outcomes | Iron deficiency anemia | ICD-10-CM D50 |
|  | Vitamin B12 deficiency anemia | ICD-10-CM D51 |
|  | Other anemia | ICD-10-CM D64 – Other anemias |
|  | All-cause hospitalization | Inpatient encounter visit types: HL7V3.0 VisitType IMP, NONAC, ACUTE |
|  | Emergency department visit | CPT 1013711 – Emergency Department Services |
| Variables included in propensity score matching | Demographics | Age, sex, race (White, Black or African American, Asian, Other race) |
|  | Comorbidities | Hypertension (I10), diabetes mellitus (E08–E13), chronic kidney disease (N18), ischemic heart disease (I20–I25), cerebrovascular disease (I60–I69), liver disease (K70–K77), chronic obstructive pulmonary disease (J44), obstructive sleep apnea (G47.33), alcohol-related disorders (F10), nicotine dependence (F17), neoplasms (C00–D49), major depressive disorder, recurrent (F33), factors influencing health status and contact with health services (Z00–Z99) |
|  | Procedure type | Sleeve gastrectomy (CPT 43775) and Roux-en-Y gastric bypass (CPT 43644) |
|  | Baseline medications | Iron, vitamin B, vitamin D |
|  | Baseline laboratory variables | Albumin, hemoglobin A1c, body mass index, hemoglobin, estimated glomerular filtration rate |

**Supplementary Table 2. Availability of Baseline Laboratory Variables and Anthropometric Variables Before and After Propensity Score Matching**

| Variable | Early cohort (Before matching) | Late cohort (Before matching) | Early cohort (After matching) | Late cohort (After matching) |
| --- | --- | --- | --- | --- |
| eGFR (MDRD) | 91.40% | 84.98% | 89.75% | 90.83% |
| Body mass index | 90.53% | 85.05% | 89.17% | 89.17% |
| Hemoglobin | 92.01% | 80.99% | 88.82% | 87.22% |
| Albumin | 88.79% | 77.39% | 84.04% | 85.27% |
| Hemoglobin A1c | 80.32% | 60.65% | 76.60% | 65.87% |

**Supplementary Table 3. Association between surgical era and 2-year postoperative outcomes before propensity score matching (90-day to 2-year window)**

| Outcomes | Late group  Events (%) | Early group  Events (%) | HR (95% CI) | Log-rank P value | Absolute risk difference |
| --- | --- | --- | --- | --- | --- |
| Nutritional anemia | 1,227 (6.33%) | 806 (5.41%) | 1.24 (1.13–1.36) | <0.001 | 0.92% |
| IDA | 990 (5.11%) | 652 (4.38%) | 1.24 (1.12–1.37) | <0.001 | 0.73% |
| B12 deficiency anemia | 203 (1.05%) | 138 (0.93%) | 1.18 (0.95–1.46) | 0.135 | 0.12% |
| Other anemia | 867 (4.47%) | 648 (4.35%) | 1.10 (0.99–1.21) | 0.078 | 0.12% |
| Hospitalization | 3,472 (17.91%) | 1,777 (11.93%) | 1.66 (1.57–1.76) | <0.001 | 5.98% |
| ED visit | 4,107 (21.19%) | 2,816 (18.91%) | 1.20 (1.14–1.26) | <0.001 | 2.28% |

HR, hazard ratio; CI, confidence interval; IDA, iron deficiency anemia; ED, emergency department.
